# Supplementary material for: Accelerating Gene Discovery by Phenotyping Whole-Genome Sequenced Multi-mutation Strains and Using the Sequence Kernel Association Test (SKAT)
Source: PLoS Genet. 2016 Aug 10;12(8):e1006235. doi: 10.1371/journal.pgen.1006235 (PMC4980031; doi:10.1371/journal.pgen.1006235)
Supplement: S7 Table — (DOCX) [file pgen.1006235.s017.docx]

**Table S7.** Guide RNA sequence and homology arm sequence used for generating CRISPR-Cas9 knockout mutations in *bgnt-1.1*

| Target | Guide Sequence | Upstream Homology Arm | Downstream Homology Arm |
| --- | --- | --- | --- |
| *bgnt-1.1* | GGGGAATGAGAACGTAAAGG | CGCATTTTAGTAGTTTCGCATTTGCATCCTTTACAGGAAAAAAAATAAATGCGAAGTTTTGGCATTTAAATTTTACATATTGTTATCAGCTATCAAGACAAATTGATTTATTTCTCAATTTTGTTTTTTTAATGCTTTGTAATTCAAAGGATGAAGCTTCCAGTGGACACTTTTAAAAGTATACGCACTGTTTTGCAAACTTTTTAATTTTTAGTTATTTCGATATTTTAATCTAAAATCTAAACTTCTTGCCTATTTAGCAGCTTCAACAACTTCTTATTCAAGTTTTGTTTAATCCCTGTTCAAACATCAAGTGTTGTATAAAATTATATGTGTGATCAAACTGAGACCACATTTTACACAAGCCTTTTTCAAAGTTGCTTAGATCTGTTGGCTCAACAAACAAAGGATTCCACCAATTCACTGTTGCCATCTGGAACCGCCTAACAAAGCTCATTCCAGAAAAAACTTAATTGTTCTGTGTTTGCTACCCGGTTAAA | AAACAAGTCAAAAGAACAATTTGTCAAATATTTCAAAAAAGTTGGTACTTTTTAATAAATTAAGAGCAATTTATAGCTCCTCCAATGGTGGTTGACTGTCATCACCTTGGATGCAATAGTTTCAGAACTTGTAAAAACTTAAATGTATTGACCAAACTTGAAAATAATGCTATACATTACCCGCCAACTTCTTGCACAACTTGAGCTCTGACATCATTCACGTTTTTCAAATTTTTCTTCAAAATTCCAAATCTAAGCAAACTTCGAGTTTTCAAATATAAAAATTGAATCTTTCTCAAACCCTTCATTTTAAAATGGAAATAATCAATAGAAACAGTTCAATCATTTTTCGATTCTTTCTCTTTCTTCTTCTTTTTCAAAATCTTCATAAACTTATTCTTAGTTCCTGGATCACTTGCTCCGGTTTCACTCATCACAGTCATACGTTCAAAGTCCCCGATTGCCATGGTCATGCTGGCTCGACAAGATGCATCAACAAA |
