# Supplementary material for: Accelerating Gene Discovery by Phenotyping Whole-Genome Sequenced Multi-mutation Strains and Using the Sequence Kernel Association Test (SKAT)
Source: PLoS Genet. 2016 Aug 10;12(8):e1006235. doi: 10.1371/journal.pgen.1006235 (PMC4980031; doi:10.1371/journal.pgen.1006235)
Supplement: S8 Table — (DOCX) [file pgen.1006235.s018.docx]

**Table S8.** Primers used to validate *bgnt-1.1* CRISPR-Cas9 knockout alleles

| CRISPR-Cas9 Validation Primer | Sequence (5’ -> 3’) |
| --- | --- |
| F01D4.9-1-L | GAAATCAGCCAGAGTCACCAAAGG |
| pMyo2-SEC | CCCTCAATGTCTCTACTTGT |
| NeoR-SEC | TTCCTCGTGCTTTACGGTATCG |
| F01D4.9-1-R | GCTCGCACCAGAAACATTGTCATC |
